# Microscopic Colitis in a Young Male: Unveiling the Rarity

**DOI:** 10.7759/cureus.51638

**Published:** 2024-01-04

**Authors:** Yumna Shahid, Zahabia Sohail, Adeel Urrehman, Zeeshan Uddin

**Affiliations:** 1 Gastroenterology, Aga Khan University Hospital, Karachi, PAK; 2 Medicine, Aga Khan University Hospital, Karachi, PAK; 3 Pathology and Laboratory Medicine, Aga Khan University Hospital, Karachi, PAK

**Keywords:** rare cause of diarrhea, irritable bowel syndrome, young adult male, sigmoidoscopy, persistent diarrhea, microscopic colitis

## Abstract

In recent decades, microscopic colitis (MC) has become increasingly recognized as a common contributor to diarrhea and lower gastrointestinal symptoms, particularly among the older demographic. The condition is distinguished by persistent diarrhea with loose watery stools, and endoscopic examination is typically normal with characteristic histopathologic findings. MC is rarely seen under 30 years of age and is less common in males. Our case highlights an exceedingly uncommon clinical setting as it involves a young male who was diagnosed with collagenous colitis. The diagnosis of MC can easily be missed by physicians during initial evaluation. Specifically in irritable bowel syndrome patients with diarrhea predominant symptoms, a colonoscopy should be performed and biopsies should be taken from the entire colon to rule out MC.

## Introduction

In recent decades, microscopic colitis (MC) has surfaced as a prevalent contributor to diarrhea and lower gastrointestinal symptoms, particularly among the older demographic. The condition is distinguished by persistent, liquid diarrhea and endoscopic examination is typically normal with characteristic histopathologic findings. The most common risk factors associated include smoking, alcohol abuse, drugs like nonsteroidal anti-inflammatory drugs (NSAIDs)(including diclofenac and naproxen) proton-pump inhibitors (PPIs)(like omeprazole, lansoprazole), and antidepressant or antipsychotic drugs (sertraline, escitalopram) [1]. MC is divided into two types lymphocytic colitis (LC) and collagenous colitis (CC). Intraepithelial lymphocytosis is seen in LC, while CC is recognized by a thickened subepithelial collagen band [2]. The occurrence of the CC is approximated to range from 2 to 10.8 cases per 100,000 annually, while the incidence of the LC falls between 2.3 and 16 cases per 100,000 per year [3]. Moreover, there is a higher prevalence observed in northern Europe and North America. The mean age at the time of diagnosis is observed to be 65 years and mostly women are affected. The disease is usually managed with lifestyle modification, antidiarrheal medication, bismuth subsalicylate, budesonide and cholestyramine [3].

In the pediatric and young adult population, the occurrence of MC is significantly less, with certain documented rates falling below 1.0 per 100,000. CC is rarely seen under 30 years of age and less commonly in males [4]. 

## Case presentation

**Figure 1:**
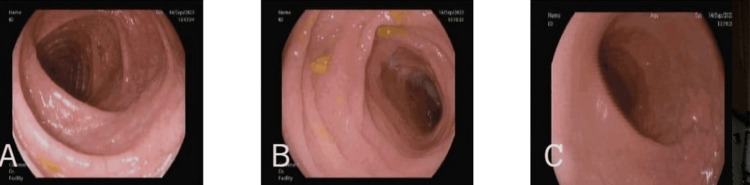
Sigmoidoscopy images showing normal-looking mucosa. Descending colon (A), Sigmoid colon (B), Rectum (C)

**Figure 2:**
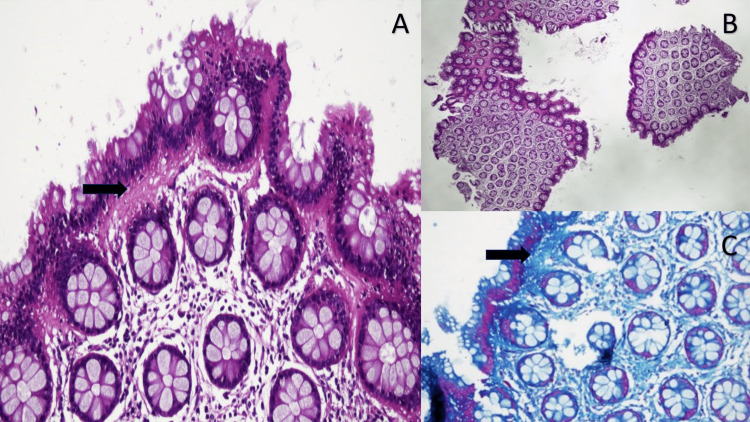
Histological appearance of collagenous colitis. (A) and (B) (H&E, 20X and 4X, respectively) show colonic mucosa with intact crypt architecture and no significant inflammation. (A) exhibits colonic mucosa with a thickened subepithelial collagen band (arrow) with entrapped inflammatory cells. (C) shows Masson trichrome special stain highlighting a thick collagen band (arrow) under the surface epithelium, consistent with collagenous colitis.

A 20-year-old male presented to the gastroenterology clinic with the chief complaint of diarrhea for 3 months. He used to have three to four episodes of diarrhea per day, small volume, with excessive mucus in stool associated with mild abdominal cramps. There was no history of fever or bleeding per rectum. No significant weight loss. There is no notable medical or surgical history, aside from intermittent use of proton pump inhibitors to address occasional dyspepsia. He used to take omeprazole 40 mg as needed. He had been taking symptomatic treatment for diarrhea on and off. There was no history of stress or anxiety-related symptoms. Upon examination, the individual exhibited stable vital signs with no postural hypotension. Noteworthy was the absence of anemia, jaundice, or clubbing. Furthermore, the abdomen was found to be soft and non-tender. His hemoglobin (Hb) was 13 g/dL, mean corpuscular volume (MCV) 91 fL, WBC count 6x10^9/L and platelets of 300x10^9/L. A stool analysis was done, which was normal. No pus cells or cysts were identified in the stool. Tissue transglutaminase antibodies IgA and IgG were checked, which were negative. Ultimately, a sigmoidoscopy was performed to ascertain the underlying cause. Sigmoidoscopy revealed normal-looking mucosa. The examination was done till the descending colon (Figure 1A). Biopsies were obtained from both the descending and sigmoid colon (Figure 1B). The histopathology report yielded a thickened subepithelial collagen band with entrapped inflammatory cells (Figures 2A-2C). These findings were indicative of microscopic colitis, with a notable emphasis on features aligning with collagenous colitis. The patient was started on oral budesonide therapy at 9 mg daily for 6 weeks and was followed up after 6 weeks. Improvement in the patient's condition became apparent, manifested by a decrease in the frequency of diarrhea episodes. No maintenance therapy was given as the patient went into remission.

## Discussion

CC and LC exhibit remarkably similar symptoms, making it challenging to discern one from the other based solely on clinical manifestations. Consequently, the distinction between these two conditions relies solely on histological analysis. The typical clinical presentation involves persistent (either recurrent or intermittent) episodes of watery, non-bloody diarrhea. It is common for symptoms to persist for several months to 2-3 years before seeking medical attention and receiving a diagnosis. The diagnosis of microscopic colitis is contingent upon: (1) a compelling clinical history with the exclusion of alternative causes; (2) endoscopic or radiographic findings that are normal or nearly normal; and (3) histopathological examination of endoscopic biopsies, revealing characteristic findings indicative of microscopic colitis [5].

The frequency of microscopic colitis has been observed to escalate with both increasing age and female gender. Primarily, women tend to be affected during their fifth and sixth decades of life [6,7]. Turner et al studied the ethnic distribution of MC and found that it is less prevalent among Indians and East Asian descent [8]. This also signifies the rarity of this case since our patient is a young male who belongs to the Indian subcontinent (Southeast Asia). Very limited data is available regarding microscopic colitis under 30 years of age (including children and young adults) [4,9,10]. The majority of the case reports and especially standardized studies that examine larger cohorts have been performed among older adults [11,12].

The diagnosis of MC can easily be missed by physicians during initial evaluation. A study by Mohamed et al also showed a few cases of microscopic colitis which were previously labeled as diarrhea-predominant irritable bowel syndrome [13]. Therefore, colonoscopy and biopsy are mandatory to establish diagnosis and rule out MC. Multiple random colonic biopsies from both the right and left colon are recommended even if the underlying mucosa looks normal [5,14]. Specifically in irritable bowel syndrome patients with diarrhea-predominant symptoms, a colonoscopy should be performed and biopsies should be taken from the entire colon to rule out MC [15,16].

Common preventive measures involve quitting smoking and refraining from the use of nonsteroidal anti-inflammatory drugs. It is advisable to discontinue any medications linked to the onset of MC whenever feasible. The primary goal of antidiarrheal therapy is to reduce the occurrence of nighttime episodes, and the preferred agent for this purpose is loperamide. Budesonide exhibits potent glucocorticoid properties, demonstrating a wide-ranging anti-inflammatory effect and a strong affinity for intracellular glucocorticoid receptors [17].

## Conclusions

This particular case underscores the importance of contemplating uncommon pathologies across various age groups and demographics. It emphasizes the necessity of not overlooking a diagnosis by forgoing biopsies, especially when the colonic mucosa appears normal. It advocates for a cautious approach, suggesting that not all instances of diarrhea should be hastily attributed to irritable bowel syndrome; rather, a thorough investigation is warranted when all diagnostic evaluations yield negative results.
